# Liver *FOXP3* and *PD1/PDL1* Expression is Down-Regulated in Chronic HBV Hepatitis on Maintained Remission Related to the Degree of Inflammation

**DOI:** 10.3389/fimmu.2013.00207

**Published:** 2013-07-25

**Authors:** Georgios Germanidis, Nikoletta Argentou, Prodromos Hytiroglou, Themistoklis Vassiliadis, Kalliopi Patsiaoura, Anastasios E. Germenis, Matthaios Speletas

**Affiliations:** ^1^First Department of Internal Medicine, AHEPA Hospital, School of Medicine, Aristotle University of Thessaloniki, Thessaloniki, Greece; ^2^Department of Immunology and Histocompatibility, Faculty of Medicine, School of Health Sciences, University of Thessaly, Biopolis, Larissa, Greece; ^3^Department of Pathology, AHEPA Hospital, School of Medicine, Aristotle University of Thessaloniki, Thessaloniki, Greece; ^4^First Propetheutic Department of Internal Medicine, AHEPA Hospital, Aristotle University of Thessaloniki, Thessaloniki, Greece; ^5^Department of Pathology, Hippokration Hospital, Thessaloniki, Greece

**Keywords:** chronic HBV hepatitis, regulatory T cells, FOXP3, PD1/PDL1, FAS/FASL, inflammation

## Abstract

**Background and Aim:** T cell expression of PD1 and inhibition of T effector cells by Foxp3^+^-T regulatory cells are among the most powerful mechanisms for achieving a balanced immune response. Our aim was to investigate, how liver *FOXP3* and *PD1/PDL1* expression is regulated in chronic HBV hepatitis (CHB) on maintained long-term remission in comparison with active disease, and whether they are correlated to the expression of pro- and anti-inflammatory cytokines and apoptosis mediators, along with the degree of histological inflammation and markers of T cell effector restoration.

**Methods:** Fifty-three HBeAg-negative CHB patients with both active (30) and completely remitted disease on long-term antiviral treatment (23) and four controls (submitted to liver biopsy due to a mild increase of aminotransferases but without liver necroinflammatory and architecture changes) were enrolled in the study. Liver mRNA levels of immunoregulatory genes (*FOXP3*, *IL10*, *TGFB1*, and those of PD1/PDL1/PDL2 pathway), major apoptosis mediators (*FAS*, *FASL*, *TNFA*, *TRAIL*), cytokines of effector T cell restoration (*IL2*, *IFNG*), and those of *IL1B*, *CD4*, and *CD8*, were evaluated by quantitative real-time reverse-transcriptase PCR and were correlated with each other, along with the intensity of liver inflammation and fibrosis staging. The expression and localization of FOXP3, PD1, PDL1, CD4, and CD8 were also assessed by immunohistochemistry.

**Results:** The expression of *FOXP3*, *IL10*, *TGFB1*, *PD1*, *PDL1*, *FASL*, and *CD8* was significantly down-regulated in the remission state. In contrast, liver expression of *IL2* and *IFNG*, along with *CD4*, *IL1B*, *TNFA*, and *FAS* did not change significantly. Moreover, *FOXP3*, *PD1*, *PDL1*, and *CD8* transcripts were positively correlated to the intensity of liver inflammation.

**Conclusion:** Our data indicate that in the CHB disease model, the immunosuppressive liver environment is down-regulated in the maintained on-treatment long-term remission state and correlates with the intensity of liver inflammation, but not liver T cell restoration.

## Introduction

The most important process for the immune control and inactivation of hepatitis B virus (HBV) infection is a robust immune response, either spontaneous or treatment induced (1, 2). However, in chronic active infection (chronic HBV hepatitis, CHB), the impaired and/or unbalanced T cell responses are unable to control viral replication but are sufficient to cause chronic liver damage. The latter is initially dependent on viral antigens expressed on hepatocytes and anti-HBV specific CD8^+^-cytotoxic T-lymphocyte (CTL) responses; afterward, the chronic liver damage is amplified by non-specific liver infiltrating cells and CD4^+^-T cell interaction pathways (1, 2).

Among the most powerful mechanisms for achieving a balanced immune response are the expression of programed death 1 (PD1) molecule by T cells (3) and the inhibition of effector T cells (Teffs) by CD4^+^-T regulatory cells (Tregs) (4). Models of viral infection have indicated that the interaction between the inhibitory receptor PD1, expressed in high levels on lymphocytes, and its ligands program cell death 1 ligand (PDL)-1 and PDL2, plays a critical role in T cell exhaustion by inducing T cell inactivation (3, 5). In CHB patients, high PD1 levels are expressed by virus-specific T cells and improvement of the T cell function has been obtained *in vitro* by inhibition of the PD1/PDL1 interaction (3, 5). Particularly, the PD1/PDL1 blockade increased CD8^+^ T cell proliferation, as well as the production of interferon-gamma (IFN-γ) and interleukin (IL)-2 production by intrahepatic lymphocytes, inducing variable levels of functional T cell restoration both in the liver and in peripheral blood, with a better functional improvement among intrahepatic T cells (5). Moreover, Tregs are important mediators of immune suppression and their presence prevents reactions against self by inducing regulatory signals to antigen presenting cells (APCs) and/or Teffs (6, 7). Their ablation increases the risk of autoimmunity (8) whilst, on the contrary, their signals could also affect non-autoreactive clones, leading to inhibition of antineoplastic, antimicrobial, antiparasitic, and antiviral immune responses (7, 9).

Previous studies have indicated that patients with chronic viral hepatitis display increased numbers of Tregs (both natural and inducible) in peripheral blood (10– 12) or liver (13, 14), which, in turn, exert a suppressive function against specific HBV- or hepatitis C virus (HCV)-Teffs *in vitro* (10– 14). Interestingly, Aoki et al. reported that the loss of natural Tregs (characterized by the constitutive expression of *FOXP3* gene) induces fatal autoimmune hepatitis (AIH) in neonatal thymectomized (NTx)-PD1^−/−^ mice, due to migration of dysregulated follicular T helper (Tfh) cells from the spleen (15). In this context, we have recently demonstrated that the *FOXP3* expression in liver is positively correlated with the intensity of liver inflammation along with a specific pattern of mRNA expression of the apoptosis mediators *FAS*, *FASL*, and *TRAIL*, irrespective of the cause of tissue damage (viral, toxic, autoimmunity), suggesting that might represent a bystander effect and not a causative event of chronic inflammation (16). Considering also that our findings were in line with the attractive view of Zheng and Rudensky (7) claiming that Tregs “have a vital role in preventing autoimmunity and pathology inflicted by uncontrolled immune responses to infections,” we suggested a comprehensive protective model of Tregs to prevent catastrophic pathology on apoptosis-induced inflammation (16).

The aim of this study was to explore whether the long-term antiviral treatment in patients with HBeAg-negative CHB affects the abovementioned model, investigating also another important apoptosis pathway implicated in Teffs dysfunction in liver, namely the PD1/PDL1. Thus, the expressions of *FOXP3*, characterizing mainly nTregs (7), as well as those of *IL10* (encodes IL-10) and *TGFB1* (encodes TGF-β1), characterizing type I (Tr1) and T helper type 3 (Th3) inducible Tregs (iTregs) (17), respectively, were examined at the same time with the PD1/PDL1/PDL2 pathway, in relation to the expression of major apoptosis mediators, namely *TNFRSF6/FAS* (encodes FAS), *TNFSF6/FASL* (encodes FASL), *TNFA* (encodes TNF-α), and *TNFSF10/TRAIL* (encodes tumor necrosis factor related apoptosis inducing ligand, TRAIL). Furthermore, the expression of the inflammatory cytokine IL-1β (encoded by *IL1B* gene) and cytokines of the immune effector T cell restoration (IL-2, encoded by *IL2* gene and IFN-γ, encoded by *IFNG* gene), together with the expression of *CD4* and *CD8* were explored. Our data provide clear evidence that in CHB HBeAg-negative disease model, the immunosuppressive liver environment is down-regulated in the maintained on-treatment long-term remission state and correlates with the intensity of liver inflammation, but not with liver T cell restoration.

## Materials and Methods

### Patients

Liver biopsy specimens obtained from 53 patients with CHB were examined; 30 were newly diagnosed and were evaluated before any treatment and 23 were on maintained continuous antiviral treatment response and remission for at least 240 weeks (5 years) with entecavir. Nine out of 30 newly diagnosed CHB patients were derived from a previous study of our group (16), since their genetic material was also available for the analysis of all genes included in this study. Considering that in Eastern Mediterranean area the HBV genotype D and HBeAg-negative serological form of CHB prevails (about 90% of affected Greek patients) (18), all the enrolled patients had the abovementioned HBV genotype. The treatment efficacy at year 5 included the biochemical response based on normalized ALT levels, and the complete virologic response defined as serum HBV DNA<169 copies/mL (29 IU/mL), namely the lower limit of quantification of the COBAS TaqMan assay (Roche Molecular Systems). None of the patients presented with co-infection with other hepatitis viruses (types A, C, D, and E) or with HIV, or was receiving any other immunomodulatory treatment during the last 6 months prior to liver sampling. HBV DNA quantification was performed with the bDNA assay V2.0 (Bayer, Siemens). A summary of the demographic, clinicopathologic, and serologic data of the analyzed CHB patients is presented in Table 1.

**Table 1:** **Clinicopathological and serological data of the patients of the study**.

	Chronic HBV hepatitis at diagnosis	Chronic HBV hepatitis on sustained remission
No	30	23
Sex (M/F)^a^	13/17	18/5
Age (median, range)	47, 21–64	52, 23–73
AST^b^ (U/μL), (median, range)	43, 17–1969	24, 15–51
ALT^c^ (U/μL), (median, range)	54, 15–1478	27, 15–49
Inflammation grade^d^
I-0^d^	0	1
I-1^d^	8	18
I-2^d^	14	4
I-3^d^	6	0
I-4^d^	2	0
Fibrosis (median, range)^d^	2.5, 0–6	2.0, 0–4
HAI score (median, range)	5.5, 1–15	2.0, 0–7
Viral load (median, range)	10^5^ Meq/mL (0.007–521)	0 Meq/mL (0–0.008)

*^a^M, male; F, female; ^b^AST, aspartate aminotransferase; ^c^ALT, alanine aminotransferase; ^d^inflammation grade (I-0: without inflammation, I-1: minimal, I-2: mild, I-3: moderate, and I-4: marked) and fibrosis stage were assessed as presented in the section of Material and Methods*.

Each liver biopsy specimen was separated into two parts. One of them was immediately fixed in 10% formalin solution for diagnostic histological examination, and the other was snap frozen and stored at −80°C until further use. Formalin-embedded sections were stained by hematoxylin-eosin and Masson’s trichrome. Two independent pathologists assessed and scored each biopsy and any discrepancy was further evaluated by an expert pathologist. The samples were blinded to the timing of biopsy and treatment assignment. Core length and number of portal tracts were taken into account to determine adequacy of biopsy specimens. Biopsy slides were graded and staged with the Ishak scoring system (19, 20). Furthermore, according to the intensity of liver inflammation in the biopsy specimens, the patients were classified as I-0 (no inflammation), I-1 [minimal inflammation, histological activity index (HAI) score 1–4], I-2 (mild inflammation, HAI score 5–8), I-3 (moderate inflammation, HAI score 9–12), and I-4 (marked inflammation, HAI score 13–18) and the latter classification was used in the statistical analysis (Table 1).

Informed consent was obtained by all participants and the study was approved by the Institutional Review Board. One of the challenges experienced in this study was the obtaining of informed consent from patients undergoing liver biopsy without a clear clinical need (patients on maintained remission), considering that they had complete virologic suppression at year 5 on continuous antiviral treatment.

### Quantitative real-time reverse-transcriptase PCR

Total RNA was isolated from stored liver samples after homogenization, using TRI (Life Technologies, Invitrogen, Thessaloniki, Greece), according to the manufacturer’s instructions. Complementary DNA (cDNA) was reversed transcribed from 1 μg of the total RNA, using a random 6-mer oligonucleotide primer (50 pmol/μL) (Roche, USA) and M-MLV reverse transcriptase (Invitrogen), according to the manufacturer’s instructions.

The mRNA levels of 15 genes, namely *FOXP3*, *IL10*, *TGFB1*, *TNFRSF6/FAS*, *TNFSF6/FASL*, *TNFSF10/TRAIL*, *PD1/PDCD1* (encodes PD1), *PDL1/PDCD1LG1* (encodes PDL1), *PDL2/PDCD1LG2* (encodes PDL2), *IL2*, *TNFA*, *IFNG*, *IL1B*, *CD4* (encodes CD4), and *CD8a* (encodes CD8) were determined in a Quantitative real-time reverse-transcriptase PCR (qRT-PCR) using SYBR-Green PCR Supermix (Invitrogen, UK), in the automated thermocycler RotorGene 6000 (Corbett Life Science, Sydney, Australia). The *B2M* gene was used as an internal control for sample normalization (reference gene). An 1/20 aliquot of the cDNA reaction product was used in duplicate qRT-PCR reactions and all measurements were averaged. Primers for the amplification of the genes *FOXP3*, *IL10*, *TGFB1*, *TNFRSF6/FAS*, *TNFSF6/FASL*, *TNFSF10/TRAIL*, *IL2*, *TNFA*, *IL1B*, *PD1/PDCD1*, and *IFNG* were commercially obtained by Qiagen (Valencia, CA, USA). The primers for the amplification of *PDL1/PDCD1LG1*, *PDL2/PDCD1LG2*, *CD4*, and *CD8a* were designed with the aid of the Oligo 6.0 software (NBI, Plymouth, MN, USA) and are presented in Table 2. Thermocycling conditions of the analyzed genes are also presented in Table 2. The efficiency of each qRT-PCR reaction ranged between 0.9 and 1.05. Relative quantification and calculation of the range of confidence were performed using the comparative ΔΔ^CT^ method, as described (21). The relative expression of each gene is presented as a multiple of the respective gene expression in a sample of a patient who underwent liver biopsy due to a mild increase of aminotransferases but without liver architecture changes (histology negative for disease; “healthy” control).

**Table 2:** **Primers and PCR conditions for the amplification of the analyzed genes**.

Gene	Primers	Sequence	PCR conditions
*FOXP3*	Forward	Commercially obtained by Qiagen, Cat No PPH00029B	95°C for 10 min, followed by 40 cycles (95°C for 15 s, 60°C for 15 s, 72°C for 15 s)
	Reverse	
*IL10*	Forward	Commercially obtained by Qiagen, Cat No PPH00572B	95°C for 10 min, followed by 40 cycles (95°C for 15 s, 58°C for 60 s)
	Reverse	
*TGFB1*	Forward	Commercially obtained by Qiagen, Cat No PPH00508A	95°C for 10 min, followed by 40 cycles (95°C for 15 s, 60°C for 60 s)
	Reverse	
*FAS*	Forward	Commercially obtained by Qiagen, Cat No PPH00141B	95°C for 2 min, followed by 40 cycles (95°C for 10 s, 55°C for 10 s, 72°C for 20 s)
	Reverse	
*FASL*	Forward	Commercially obtained by Qiagen, Cat No PPH00142B	95°C for 2 min, followed by 40 cycles (95°C for 10 s, 55°C for 10 s, 72°C for 30 s)
	Reverse	
*TRAIL*	Forward	Commercially obtained by Qiagen, Cat No PPH00242E	95°C for 2 min, followed by 40 cycles (95°C for 10 s, 55°C for 10 s, 72°C for 20 s)
	Reverse	
*PD1*	Forward	Commercially obtained by Qiagen, Cat No PPH13086E	95°C for 10 min, followed by 40 cycles (95°C for 15 s, 60°C for 60 s)
	Reverse	
*PDL1*	Forward	GGTGGTGCCGACTACAA	95°C for 2 min, followed by 40 cycles (95°C for 10 s, 58°C for 10 s, 72°C for 20 s)
	Reverse	TAGCCCTCAGCCTGACAT	
*PDL2*	Forward	CTGTGGCAAGTCCTCATA	95°C for 2 min, followed by 40 cycles (95°C for 30 s, 55°C for 30 s, 72°C for 30 s)
	Reverse	TAAAGCTGCTATCTGGTGA	
*IL2*	Forward	Commercially obtained by Qiagen, Cat No PPH00172B	95°C for 10 min, followed by 40 cycles (95°C for 15 s, 60°C for 60 s)
	Reverse	
*TNFA*	Forward	Commercially obtained by Qiagen, Cat No PPH00341E	95°C for 10 min, followed by 40 cycles (95°C for 15 s, 60°C for 60 s)
	Reverse	
*IFNG*	Forward	Commercially obtained by Qiagen, Cat No PPH00380B	95°C for 10 min, followed by 40 cycles (95°C for 10 s, 58°C for 10 s, 72°C for 30 s)
	Reverse	
*CD4*	Forward	CATCAAGGTTCTGCCCACAT	95°C for 2 min, followed by 40 cycles (95°C for 10 s, 58°C for 10 s, 72°C for 20 s)
	Reverse	TTCTAAACCGGTGAGGACAC	
*CD8a*	Forward	GCTGGACTTCGCCTGTGATA	95°C for 2 min, followed by 40 cycles (95°C for 10 s, 55°C for 10 s, 72°C for 60 s)
	Reverse	TGTCTCCCGATTTGACCAC	
*B2M*	Forward	Commercially obtained by Qiagen, Cat No PPH01094E	95°C for 10 min, followed by 40 cycles (95°C for 15 s, 60°C for 60 s)
	Reverse	

### Immunohistochemistry

Immunohistochemical stains for FOXP3, PD1, and PDL1 proteins, as well as CD4 and CD8 antigens, were performed on 4 μm-thick paraffin sections of 15 newly diagnosed before any treatment and 12 on maintained continuous antiviral treatment response and remission biopsy specimens. The primary monoclonal antibodies utilized for immunohistochemistry and their dilutions are shown in Table 3. All immunohistochemical stains except for PD1 were performed in an automated Bond system (Menarini), with the use of the Bond polymer refine detection kit. Stains for PD1 were performed in a DAKO autostainer, with the use of an Envision Flex Plus kit.

**Table 3:** **Antibodies and dilutions used in the present immunohistochemical study**.

Antigen	Antibody (clone)	Dilution	Manufacturer
FOXP3	ab22510	1:50	Abcam (Cambridge, UK)
PD1	ab52587	1:25	Abcam (Cambridge, UK)
PDL1 (CD274)	29E.2A3	1:30	Biolegend (Athens, Greece)
CD4	NCL-L-CD4-1F6	1:20	Novocastra (Athens, Greece)
CD8	C8/144B	1:50	DAKO (Athens, Greece)

### Statistical analysis

For basic statistical calculations, all gene expression levels were treated as continuous variables. Differences of gene expression between different disease statuses were analyzed by the non-parametric Mann–Whitney *U* test. The association of the above parameters with inflammation and fibrosis staging was tested with the Kruskal–Wallis *H* test. Spearman’s rank correlation coefficient was used to estimate the correlations of the expression among the aforementioned genes, as well as the correlations of gene expressions with aminotransferases levels or viral load. Mann–Whitney *U* test, Kruskal–Wallis *H* test, and Spearman’s correlation analyses were appropriately performed by the using of SPSS (version 18.0, Chicago, IL, USA). Differences were considered statistically significant when the *p*-value (two sided) was <0.05.

## Results

### Gene and protein expression in relation to CHB status

**Figure 1:**
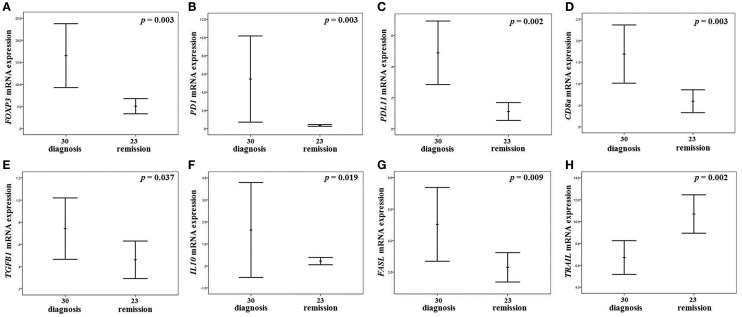
**Gene expressions with significant alteration of mRNA levels in the liver of CHB patients**. Error bar diagrams presenting the expression of *FOXP3* (**A**), *PD1/PDCD1* **(B)**, *PDL1/PDCD1LG1* **(C)**, *CD8a* **(D)**, *TGFB1* **(E)**, *IL10* **(F)**, *FASL* **(G)**, and *TNFSF10/TRAIL* **(H)** in the liver of patients in maintained on-treatment long-term remission as compared to CHB patients at diagnosis. The charts describe the algorithms for error bar computation of the mean ± 2 standard errors for the relative expression of each gene. *p*-Values in each diagram refers to Mann–Whitney *U* test.

As shown in Figure 1, patients maintained on-treatment at 5 years remission (virologic, biochemical, and histochemical) of CHB had significantly decreased mRNA levels of *FOXP3*, *IL10*, *TGFB1*, *TNFSF6/FASL*, *PD1/PDCD1*, *PDL1/PDCD1LG1*, and *CD8a*, as well as significantly increased levels of *TNFSF10/TRAIL*, as compared to patients at diagnosis with active disease. The expression levels of *IL2* and *IFNG* were also decreased, but these alterations did not reach statistical significance (Table 4). Interestingly, the alteration of *FOXP3* expression was not accompanied by a commensurate decrease of *CD4* mRNA levels.

**Table 4:** **Relative expression of the examined genes with no statistical significance between patients at diagnosis (*n* 30) and at remission (*n* 23) of the disease**.

No	Gene	CHB – diagnosis (mean ± SDEV)	CHB – remission (mean ± SDEV)	p-Value*
1	*TNFRSF6/FAS*	1.8 ± 0.9	1.8 ± 0.9	0.747
2	*PDL2/PDCD1LG2*	0.3 ± 0.2	0.2 ± 0.2	0.394
3	*IL2*	63.5 ± 226.9	7.0 ± 6.2	0.647
4	*TNFA*	35.9 ± 100.1	22.7 ± 36.7	0.342
5	*IFNG*	11.0 ± 22.8	4.0 ± 4.8	0.083
6	*IL1B*	0.6 ± 1.4	0.1 ± 0.1	0.083
7	*CD4*	0.7 ± 1.1	0.5 ± 0.5	0.628

*CHB, chronic HBV hepatitis; SDEV, standard deviation*.

*p-Values refer to Mann–Whitney U test.

**Figure 2:**
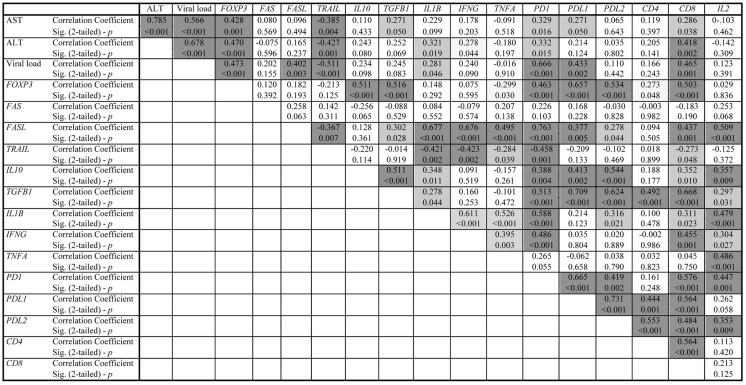
**Correlation data of chronic HBV hepatitis patients**. The dark gray shadow refers to correlation significance *p* < 0.01 (two-tailed), while the light gray shadow refers to correlation significance *p* < 0.05 (two-tailed).

The correlation of the expression between the analyzed genes, the liver biochemistry [alanine aminotransferase (AST) and aspartate aminotransferase (ALT) levels], and the viral load are presented in detail in Figure 2.

**Figure 3:**
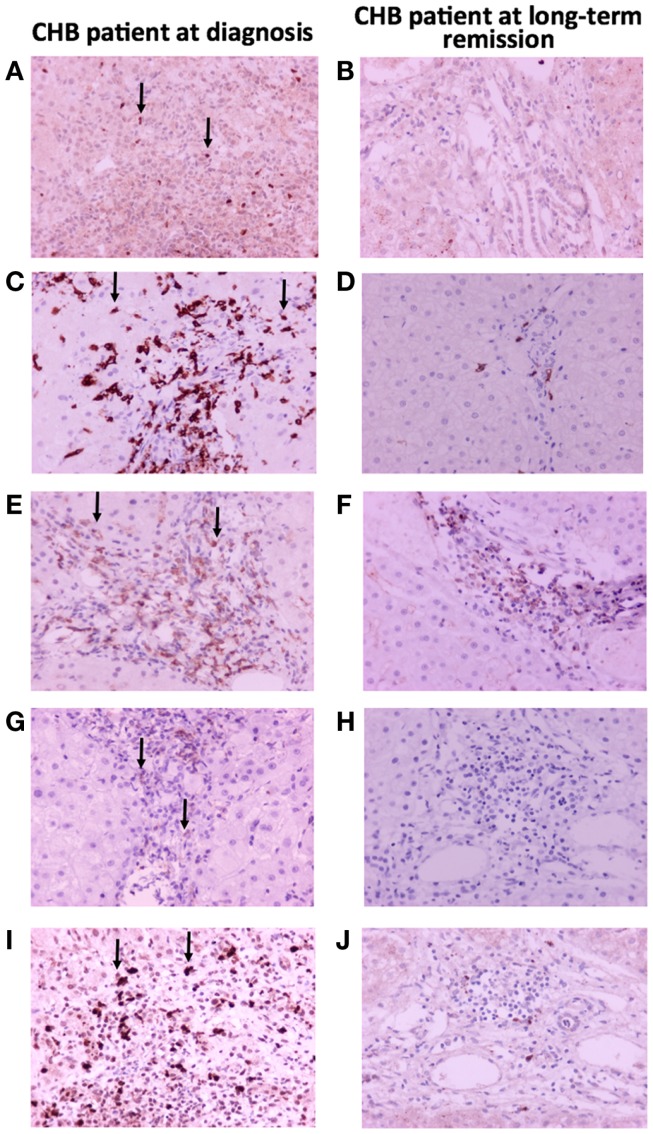
**Immunohistochemical findings in liver biopsy specimens from a patient with CHB with marked necroinflammatory activity and a patient on maintained long-term remission**. **(A,B)** FOXP3 immunopositivity in occasional lymphocytes; **(C,D)** CD8 antigen immunopositivity in many lymphocytes located in portal tracts and hepatic lobules before treatment, contrasted with rare positive lymphocytes after treatment; **(E,F)** CD4 antigen immunopositivity in some lymphocytes located in portal tracts; **(G,H)** PD1 immunopositivity in occasional lymphocytes; **(I,J)** PDL1 immunopositivity in several lymphocytes.

The immunohistochemical staining for FOXP3, PD1, and PDL1 showed small numbers of positive lymphocytes in untreated livers, while positive cells practically disappeared following treatment (Figure 3). Moreover, CD4^+^-lymphocytes were mostly located in portal tracts, while CD8^+^-lymphocytes were found in portal tracts, limiting plates, and lobules, in an extent commensurate with their nature as effectors of necroinflammatory activity (Figure 3).

### Gene expression in relation to the intensity of inflammation and the degree of fibrosis

**Figure 4:**
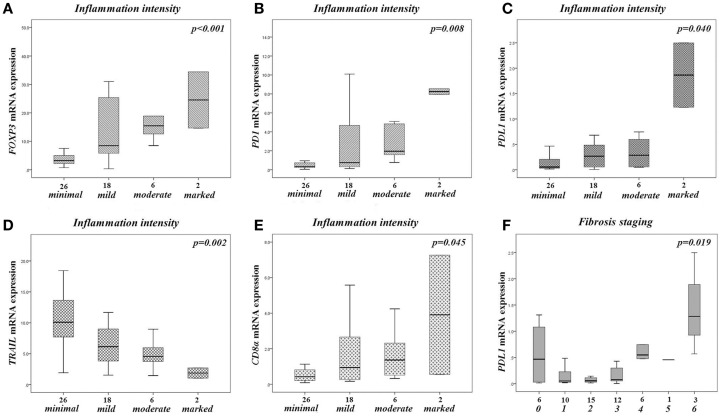
**Gene expressions in the liver of all CHB patients, according to the intensity of liver inflammation and fibrosis**. Boxplot diagrams presenting the expression of *FOXP3* **(A)**, *PD1/PDCD1* **(B)**, *PDL1/PDCD1LG1* **(C)**, *CD8a* **(D)**, *TNSF10/TRAIL* **(E)** according to the intensity of liver inflammation (excluding a sole patient with HAI score 0), and the expression of *PDL1/PDCD1LG1* **(F)** according to the severity of fibrosis (classification as presented in *Materials and Methods*). The boxes represent the interquartile range, which contains the 50% of values. The whiskers are lines that extend from the box to the highest and lowest values, excluding outliers. A line across the box indicates the median value for each patient cohort. *p*-Values in each diagram refers to Kruskal–Wallis *H* test.

In relation to the intensity of inflammation, *FOXP3*, *PD1/PDCD1*, *PDL1/PDCDLG1*, and *CD8a* exhibited a statistically significant increase of expression from minimal to marked inflammation (Figure 4). This pattern of expression was nearly similar for *TNFSF6/FASL*, although not reaching statistical significance (*p* = 0.128). On the other hand, *TNFSF10/TRAIL* displayed an opposite pattern of expression, decreasing from minimal to severe inflammation (Figure 4). The expression of the other analyzed genes was not affected by inflammation intensity (*p* > 0.05, in all cases).

The severity of fibrosis was significantly associated only with the expression of *PDL1/PDCDLG1*. A similar pattern was observed for *FOXP3* and *PD1/PDCD1*, and an opposite one for *TNFSF10/TRAIL*, although not reaching statistical significance (*p* = 0.105, *p* = 0.080, *p* = 0.060, respectively). The expression of the other analyzed genes was not affected by the severity of fibrosis (*p* > 0.05, in all cases).

Finally, as expected, HAI score was positively correlated with the fibrosis stage (*p* < 0.001, *r* = 0.665), while the viral load was also positively correlated with both HAI score and fibrosis stage (*p* < 0.001, *r* = 0.724, and *p* = 0.003, *r* = 0.403, respectively).

## Discussion

Our study provides clear evidence that in the CHB HBeAg-negative disease model, the expression of *FOXP3*, characterizing mainly nTregs, as well as those of *IL10* and *TGFB1*, characterizing Tr1 and Th3 iTregs (6), are down-regulated in the liver in the maintained on-treatment long-term remission state, as compared with cases histologically, biochemically, and virologically active at diagnosis, before any treatment. In addition, mRNA levels of liver *FASL* and *PD1* (mainly expressed by CTLs, characterized also by the expression of *CD8*), and *PDL1* (mainly attributed to infected hepatocytes and infiltrating lymphocytes) are concomitantly down-regulated in the maintained long-term remission state. However, the down-regulation of *CD8*, with no up-regulation of IL-2 (encoded by *IL2*) and IFN-γ (encoded by *IFNG*), is not in favor of restoration of T cell immune-responsiveness, but rather indicates reduction of CTLs and hepatocyte cytolysis when liver inflammation subsides on long-term antiviral treatment. These findings are also supported by our immunohistochemical findings (Figure 3). As mentioned above, the decrease of *FOXP3* expression was not followed by a commensurate decrease of *CD4* mRNA levels in human liver tissues. Obviously, this may reflect that not only Tregs are CD4^+^ but also other T cell subtypes, such as Th17 cells (6). However, a more specific analysis of T cell subpopulations by FCM was not available in our human liver tissues, and this is one of the limitations of our study. Consequently, the alterations of the frequency of CD4^+^-T cells identified in our study could not confidently be attributed to a specific T cell subpopulation.

Our data further support the notion that the PD1/PDL1 pathway (elevated levels of PD1 on T cells and increased expression of PDL1 on hepatocytes) is associated with T cell dysfunction in chronic HBV and HCV infections (3). In this context, it has been suggested that the disruption of this pathway is a logical therapeutic strategy to rescue the dysfunctional T cells, aiming to restore HBV/HCV-specific T cell responses. Fisicaro et al. (5) have also reported in short term experimental *ex vivo* CHB models that the functional recovery of HBV-specific T cells following PD1/PDL1 blockade was more pronounced for liver-resident T cells rather than peripheral T cells, and was characterized by CD8^+^ cell proliferation and the production of IFN-γ and IL-2 by intrahepatic lymphocytes. However, it is still uncertain whether the expression of PDL1 on hepatocytes truly contributes to the development of T cell exhaustion or if it is a homoeostatic mechanism that dampens the inflammatory reaction (3). Kassel et al. reported that the hepatic expression of PD1/PDL1 molecules links more directly with the degree of inflammation than with the underlying etiology of liver damage, concluding that the PD1 pathway may assist the liver in protecting itself from immune-mediated destruction (22). Accordingly, our findings did not support an antiviral Teffs function restoration in long-term maintained remission of chronic HBV infection, since no significant differences of the expression of *CD4*, *IL2*, and *IFNG* were observed. Interestingly, the abovementioned findings, considering Teffs function at remission, are in line with the findings of Nan et al. suggesting that the impaired immune responses of CHB patients are not fully restored by therapy, since no significant differences in the expression of IFN-gamma were found (23). On the other hand, the expressions of *PD1* and *PDL1* were significantly associated with the intensity of histological liver inflammation. Thus, we further support the conclusions of Kassel et al. suggesting that the down-regulation of PD1 and PDL1 molecules on maintained remission represents an epiphenomenon, contributing to, or resulting from, the resolution of an active liver inflammation.

Furthermore, we observed a down-regulation of the apoptosis mediators *FAS* and *FASL* in the maintained long-term remission state in CHB patients. Considering that previous studies, including ours, have demonstrated that the contribution of Fas/FasL pathway in CHB is of utmost importance, closely related to the degree of liver inflammation (16, 24), our findings further confirm the notion that it represents the most common and efficient pathway to kill virally infected cells in liver (25). On the other hand, we unexpectedly observed an inverse correlation of *TRAIL* expression with the intensity of liver inflammation and the disease stage (active vs. remission), since patients on maintained remission displayed an up-regulation of its mRNA levels in liver. TRAIL is a newly characterized TNF family member, triggering apoptosis in various tumor and virus-infected cells, by binding to certain death receptors, namely DR4 and DR5 (26–28). However, TRAIL can also bind to the decoy receptors DcR1, neutralizing its downstream effect, and DcR2 causing activation of NFkappaB, leading to transcription of genes known to antagonize the death-signaling pathway and/or to promote inflammation (29, 30). As a result, the increased levels of TRAIL are capable of not only inducing apoptosis but also reducing inflammation, as it has already been shown in a rabbit knee model of inflammatory arthritis (31). Considering that we have not investigated the activation cascades of TRAIL in our disease model, further studies are required in order to shed light on the precise role this protein plays in the pathogenesis and/or restoration of liver inflammation.

Likewise, we observed a significant reduction of mRNA levels of genes, which are indicative of T cell mediated immunosuppression, namely *FOXP3*, *IL10*, and *TGFB1*. Moreover, the expression pattern of *FOXP3* was identical with those observed by *PD1* and *PDL1* genes, characterized by a significant positive correlation with the intensity of liver inflammation (Figure 4). Although, Foxp3^+^-Tregs seemed to protect the liver from immune damage and compromise virus control during acute experimental HBV infection (5, 32), their role in chronic viral infections, both HBV and HCV, has been shown to range from suppressing T cell responses directed against viruses to down-regulating the immune responses causing the liver damage (5). Thus, the initial expansion stage of the adaptive immune response against viruses is followed by a contraction stage, during which Tregs might play a prominent role in maintaining a delicate balance between a robust immune response to clear the infection and the immunopathological consequences of sustained immune activation and inflammation (5).

Furthermore, recent data suggest that CD4^+^CD25^+^-Tregs play an active role in CHB not only in modulating effectors of immune response to HBV, but also in influencing the disease prognosis. Several groups have reported that the frequency of Foxp3^+^-Tregs in liver is significantly increased in patients with severe CHB compared to healthy controls (1, 5, 10–14, 33, 34), while their frequency in peripheral blood is significantly correlated with serum viral load (13, 33). Interestingly, in such patients the depletion of circulating Tregs led to an increase of IFN-γ production by HBV-Ag-stimulated peripheral blood mononuclear cells (PBMC). In addition, CD4^+^CD25^+^-Tregs were capable of suppressing the proliferation of autologous PBMC mediated by HBV antigens, probably reflecting the generation of HBV-Ag-specific tissue and circulating Tregs (13). In this context, Stross et al. have recently demonstrated that Tregs significantly delayed the clearance of HBV from blood and infected hepatocytes in a mouse model of acute HBV infection, by down-regulating antiviral activity of Teffs through limiting cytokine production and cytotoxicity (32).

However, we recently observed that accumulation of Foxp3^+^-Tregs takes place in patients with chronic liver inflammation independently of the initial inducer of liver injury (toxic, autoimmunity, and viral, including HBV infection), and it is correlated with elevated expression of apoptosis mediators FAS, FASL, and TRAIL (16). As a result, we have suggested a protective role of Tregs expansion in chronic liver inflammation, in order to prevent self-tissue damage and to avoid catastrophic pathology (16). Should this be the case, the described suppression of virus-specific T cells could be considered as a bystander effect of the nTregs that have been expanded due to the persistent apoptosis-induced inflammation. In favor to our hypothesis, Peiseler et al. have recently reported the presence of normal frequencies and function of Tregs in patients with type 1 AIH; indeed, they found higher Treg frequencies in blood and liver tissue during active disease, correlated with the inflammatory activity of the liver, compared with remission (35). Moreover, Otano et al. have recently demonstrated an increase of hepatic Tregs accompanied by a significantly high expression of anti-inflammatory cytokines, such as TGF-β1 and IL-10, and immunosuppressive molecules, such as PD1/PDL1, in WHV-chronically infected woodchucks (36). Thus, similarly to chronic HBV infection, persistent WHV infection is associated with a strong immunosuppressive environment within the liver. We consider that the results presented herein, including the study of PD1/PDL1 pathway, although correlative rather than conclusive, further support the abovementioned proposed model.

As mentioned above, our CHB HBeAg-negative patients on maintained on-treatment long-term remission displayed a down-regulation of the hepatic expression of *FOXP3*, *PD1*, and *PDL1* that was also correlated with a minimal intensity of liver inflammation. However, these patients did not exhibit immune restoration phenomena, as they are evident in the *ex vivo* human HBV infection (5, 37) and the animal models of acute (32) and chronic liver viral infections (36). Therefore, the targeting of Tregs and/or PD1/PDL1 pathway in the acute, or the early chronic HBV infection setting, should be carefully considered as a therapeutic strategy, since their depletion may trigger autoimmune phenomena or increase immune-mediated liver damage.

In conclusion, our data indicate that in the CHB HBeAg-negative disease model, the immunosuppressive liver environment is down-regulated in the maintained on-treatment long-term remission state, as compared with cases histologically, biochemically, and virologically active at diagnosis, before any treatment. In addition, the contraction of the inhibitory pathways, as measured by the down-regulation of their liver mRNA expression in long-term remission, is possibly a mere consequence of the diminution of liver inflammation, after being hyper-expressed, in order to counterbalance excessive allo- and/or auto-reactive Teffs clones.

## Conflict of Interest Statement

The authors declare that the research was conducted in the absence of any commercial or financial relationships that could be construed as a potential conflict of interest.
